# Intercellular Communication Reveals Therapeutic Potential of Epithelial-Mesenchymal Transition in Triple-Negative Breast Cancer

**DOI:** 10.3390/biom12101478

**Published:** 2022-10-14

**Authors:** Yang Liu, Yu Fang, Lili Bao, Feng Wu, Shilong Wang, Siyu Hao

**Affiliations:** 1 Pharmacy Intravenous Admixture Services, The Second Affiliated Hospital of Harbin Medical University, Harbin 150081, China; 2 Department of Phase I Clinical Trial Ward, Harbin Medical University Cancer Hospital, Harbin 150081, China; 3 Department of Gastroenterology, The First Affiliated Hospital of Harbin Medical University, Harbin 150001, China; 4 Department of Dermatology, The Second Affiliated Hospital of Harbin Medical University, Harbin 150081, China

**Keywords:** single-cell transcriptomics, triple-negative breast cancer, epithelial-mesenchymal transition, cell communication, anti-cancer strategy

## Abstract

(1) Background: Triple-negative breast cancer (TNBC) is an aggressive subtype of breast cancer with high intra-tumoral heterogeneity. The epithelial-mesenchymal transition (EMT) is one of the inducers of cancer metastasis and migration. However, the description of the EMT process in TNBC using single-cell RNA sequencing (scRNA-seq) remains unclear. (2) Methods: In this study, we analyzed 8938 cellular gene expression profiles from five TNBC patients. We first scored each malignant cell based on functional pathways to determine its EMT characteristics. Then, a pseudo-time trajectory analysis was employed to characterize the cell trajectories. Furthermore, CellChat was used to identify the cellular communications. (3) Results: We identified 888 epithelium-like and 846 mesenchyme-like malignant cells, respectively. A further pseudo-time trajectory analysis indicated the transition trends from epithelium-like to mesenchyme-like in malignant cells. To characterize the potential regulators of the EMT process, we identified 10 dysregulated transcription factors (TFs) between epithelium-like and mesenchyme-like malignant cells, in which overexpressed forkhead box protein A1 (*FOXA1*) was recognized as a poor prognosis marker of TNBC. Furthermore, we dissected the cell-cell communications via ligand-receptor (L-R) interactions. We observed that tumor-associated macrophages (TAMs) may support the invasion of malignant epithelial cells, based on CXCL-CXCR2 signaling. The tumor necrosis factor (TNF) signaling pathway secreted by TAMs was identified as an outgoing communication pattern, mediating the communications between monocytes/TAMs and malignant epithelial cells. Alternatively, the TNF-related ligand-receptor (L-R) pairs showed promising clinical implications. Some immunotherapy and anti-neoplastic drugs could interact with the L-R pairs as a potential strategy for the treatment of TNBC. In summary, this study enhances the understanding of the EMT process in the TNBC microenvironment, and dissections of EMT-related cell communications also provided us with potential treatment targets.

## 1. Introduction

Breast cancer is the most common cancer among women and is one of the causes of mortality for women worldwide [1]. Across all of the subtypes of breast cancer, triple-negative breast cancer (TNBC) represents up to 20% of all breast cancers and shows the least favorable outcomes in patients. TNBC is defined as the breast cancer subtype, which lacks expression of the estrogen receptor (ER), progesterone receptor (PR), and human epidermal growth factor receptor 2 (HER2) proteins [2,3]. In the past few years, tumor microenvironment heterogeneity played an important role in the progression of multiple cancer types [4,5]. Notably, accumulating studies have revealed the correlation between TNBC and microenvironmental heterogeneity [6,7,8]. Therefore, dissection of the TNBC microenvironment will facilitate the development of novel cancer treatment strategies.

The epithelial-mesenchymal transition (EMT) process is one of the important pathways of tumor invasion and metastasis [9,10]. Its molecular driving mechanism in TNBC continues to be revealed; for example, Hagi et al. have shown that *miR-34a-5p* restoration can suppress the epithelial-mesenchymal transition (EMT) process and inhibit the TGF-β singling pathway in MDA-MB-231 cells [11]. Not coincidentally, several studies have also shown the contribution of the EMT process to TNBC progression and metastasis [12,13]. These highlighted the importance of the EMT process in TNBC development.

The advent of single-cell RNA sequencing (scRNA-Seq) has allowed researchers to characterize intercellular heterogeneity at the single-cell level [14,15]. However, the utilization of scRNA-seq to delineate the crosstalk between EMT and tumor microenvironment is still limited in TNBC. Traditional bulk RNA-seq calculates the average expression level of tissue. Variation in individual gene expression does not localize to specific cell types and cellular regulatory mechanisms. Although multiple studies reveal the molecular subtypes and pathogenesis of breast cancer based on bulk RNA-seq [16,17,18], high-resolution exploration of molecular mechanisms is lacking.

In this study, we employed the single-cell transcriptome to characterize the microenvironment heterogeneity in five TNBC patients. The EMT process in TNBC development was revealed by defining epithelial-like and mesenchyme-like malignant cells. In addition, we identified twelve dysregulated EMT-related TFs, in which overexpressed *FOXA1* and *MSX2* were recognized as poor prognosis markers of TNBC. Further analysis of intercellular communications showed that monocytes/TAMs could interact with epithelial-like and mesenchymal-like malignant cells. We also found that TNF signaling molecules secreted by monocytes/TAMs could play an important role in mediating tumor cell metastasis. 

## 2. Materials and Methods

### 2.1. Data Acquisition

Single-cell sequencing data and patient information for TNBC were obtained from the Gene Expression Omnibus (GEO) database (GSE148673 [19]), which included five triple-negative breast cancer samples from five patients. The annotation information of the human genome (GRCh38), including the location information of the gene, was obtained from GENCODE [20] to quantify the copy number variation (CNV). Moreover, Hallmark, KEGG, and GO Biological Process gene sets were collected from the MSigDB (v6.2) for the gene set enrichment analysis and pathway signal score calculation. 

### 2.2. Clustering and Annotation of Cell Types

First, we used the R package Seurat [21] to integrate the single-cell expression matrix and cell information. Quality control was used to screen high-quality single cells, which is defined as at least 200 genes detected and the percentage of mitochondrial genes is less than 5%. Finally, 8938 cells were selected for the next analysis. Further, feature selection and dimension reduction were performed by Seurat. The normalized expression matrix was subjected to principal component analysis (PCA), and the first 20 principal components were used to perform Louvain clustering of cells with a resolution parameter of 0.2. Considering the possible batch effect between samples, the R package Harmony was used to correct the effect of donors. For each cluster of cells, we collected cell markers of breast tissue from the previous literature [22] to define the cell type of each cluster. Moreover, further re-clustering iterations (20 PCs, resolution parameter range 0.2–0.3) were performed in multiple cell types (including T cells and myeloid cells) to identify cell subtypes and states.

### 2.3. Analysis of Single-Cell Copy Number Variant

We assessed the copy number variant (CNV) for individual cells with the R package inferCNV (https://github.com/broadinstitute/inferCNV, accessed on 5 September 2022), which is designed to infer copy number instability from tumor scRNA-seq data. In brief, inferCNV compares the expression levels of genes across malignant cells to those in normal cells. The subsets of T cells, B cells, and myeloid cells were used as a reference, while sex chromosomes were excluded.

### 2.4. Gene Set Level Analysis

The gene sets related to EMT were collected from MSigDB [23]. To calculate the single-cell signature score of the gene sets, the R software package Vision (v2.0.0) [24] was used, which is a tool for annotating the sources of variation in single-cell RNA-seq data in an automated and scalable manner. Wilcoxon rank-sum test with Benjamini-Hochberg FDR correction was used to determine the enrichment of differential signature scores between groups. Cancer cells with an EMT signature score of GO Biological Process < −0.02 and an EMT signature score of Hallmark < −0.18 were defined as epithelial-like cells, and cancer cells with an EMT signature score of GO Biological Process > 0.1 and an EMT signature score of Hallmark > 0.08 were defined as mesenchymal-like cells. The cutoff is defined as the upper and lower quartiles of the EMT signature score [25]. Differential expression analysis comparing cells from epithelial-like cells and mesenchymal-like cells was performed using the FindMarkers function of Seurat. Gene set enrichment analysis (GSEA) was performed using the R package clusterProfiler [26] on gene sets sorted by log2 (fold change). Gene sets with *p* < 0.05 were considered significant.

### 2.5. Trajectory Inference Analysis

For cancer cells, the R package Slingshot [27] (v1.6.1) was used to calculate the pseudotime trajectory of epithelial cell differentiation. Cancer cells from five patients were re-clustered, resulting in nine clusters. Notably, epithelial-like cells were enriched in cluster 0. Further, we considered the cluster 0 cells as the root state and then calculated the trajectory and pseudotime. For the estimated pseudotime of each cell, we performed a two-dimensional visualization on the t-SNE of the cancer cell. 

### 2.6. Construction of the Transcriptional Regulatory Network

First, the Pearson correlation algorithm was used to identify genes related to EMT trajectories, based on 3633 time points. We set the relationship with a *p*-value < 0.05 and correlation coefficient |R| > 0.5 to be significantly correlated. Among genes related to EMT trajectory, transcription factors (TFs) were considered to be the driving factor of transcription imbalance. Human TFs data were collected from AnimalTFDB (v3.0) [28]. The TF-target gene relationship in the EMT process was constructed based on experimentally confirmed transcriptional regulatory pairs collected from TRRUST [29] and ORTI [30].

### 2.7. Cell Communication Analysis Using CellChat

We identified the intercellular communications via ligand-receptor interactions. The analysis was conducted by the R package CellChat [31]. We first required that all ligand-receptor pairs were expressed in the 15 cell subpopulations, and built a ligand-receptor sub-network, which was saved in the human ligand-receptor pair database CellChatDB. Further, we inferred a biologically significant cell-cell communication network mediated by ligand-receptor interactions. Moreover, interacting ligand-receptor pairs belonging to the TNF, ANGPTL, CXCL, and IL families were selected to assess the relationship between cell types.

## 3. Results

### 3.1. Single-Cell Transcriptome Depicts TNBC-Associated Cell Populations

**Figure 1:**
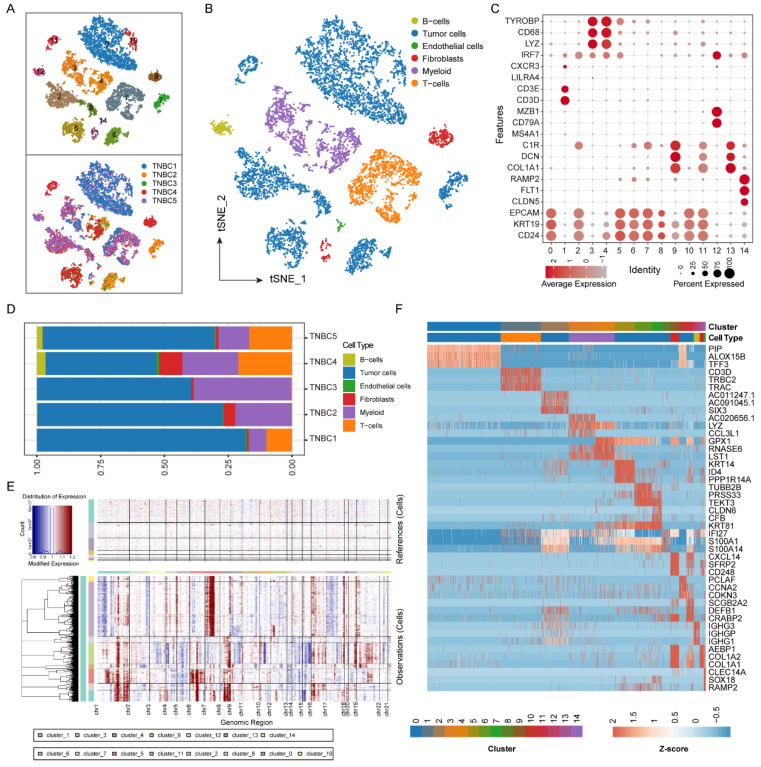
Cellular composition of TNBC and identification of malignant cells. (**A**,**B**) The t-SNE visualization of 8938 cells identified by scRNA-seq across five TNBC tumors. Clusters were marked for the top of (**A**) and patients’ information were marked for the bottom of (**A**). The cell types were annotated for (**B**). (**C**) Average expression of gene markers of each cell type. (**D**) Relative proportions of cell types for each TNBC patient. (**E**) Heatmap showing the inferred CNA profiles for reference and malignant cells. Chromosomal amplification and deletion were colored by red and blue, respectively. Each row represents a single cell, and each column denotes a chromosomal position. (**F**) Heatmap showing the expression levels of top three gene markers for each cell clusters.

In order to understand the heterogeneity of the intertumoral microenvironment, we collected scRNA-Seq data of tumor tissues from five TNBC patients diagnosed with ER/PR/HER2 negative status from the MD Anderson Cancer Center (GSE148673 [19]). After quality control and normalization, a total of 8938 cells among all the patients were employed in the subsequent analysis (Figure S1). By applying the t-distributed stochastic neighbor embedding (t-SNE) clustering algorithm (Figure S2), we obtained fifteen cell clusters (Figure 1A, cluster 0–14 in the top panel). Tumor cells (cluster 0, 2, 5, 6, 7, 8, 10, and 11) were distinguished by the expression of *CD24*, *KRT19*, and *EPCAM* [22]. Cell-specific marker genes were utilized to further determine the distribution of most expected stromal and immune cell types, including fibroblasts (cluster 9 and 13) by marker genes *COL1A1*, *DCN*, and *C1R*; endothelial cells (cluster 14) by marker genes *CLDN5*, *FLT1*, and *RAMP2*; T cells (cluster 1) by marker genes *CD3D* and *CD3E*; B cells (cluster 12) by marker genes *CD79A* and *MZB1*; and myeloid cells by marker genes *LYZ*, *CD68*, and *TYROBP* (cluster 3 and 4) (Figure 1B,C). In addition, we compared the proportion of each cell type across our patients. There were obvious individual differences among the composition of the microenvironment in TNBC patients (Figure 1A,D). An excessive proportion of malignant epithelial cells may be a bias in the sampling process. 

### 3.2. Subclonal Heterogeneity Defines Malignant Epithelial Cells of TNBC

Since breast cancer is largely driven by the altered DNA copy number [32], we estimated single-cell CNV profiles using inferCNV to distinguish malignant from normal cells (Materials and Methods). We further confirmed that the malignant cells have more copy number expansion signatures and deletions compared to other stromal cells (reference cells) (Figure 1E). Additionally, to better characterize the differences in gene expression between malignant cells and all other cells, we identified the top three genes with the highest expression intensities in each cell population (Figure 1F). A total of 15 genes were recognized in the malignant epithelial cells of TNBC (*PIP*, *ALOX15B*, *TFF3*, *SIX3*, *AC011247.1*, *AC091045.1*, *KRT14*, *ID4*, *PPP1R14A*, *TUBB2B*, *PRSS33*, *TEKT3*, *CLDN6*, *CFB*, *CXCL14*, *SFRP2*, *CD248*). Notably, 8/15 genes (*SFRP2*, *CXCL14*, *CD248*, *ALOX15B*, *KRT14*, *SIX3*, *KRT14*, *CLDN6*) were related to the epithelial-mesenchymal transition (EMT) process reported by the previous studies [33,34,35,36], highlighting that the EMT process might play an important role in TNBC development. 

### 3.3. Heterogeneity in Tumor-Infiltrating T-Cells

**Figure 2:**
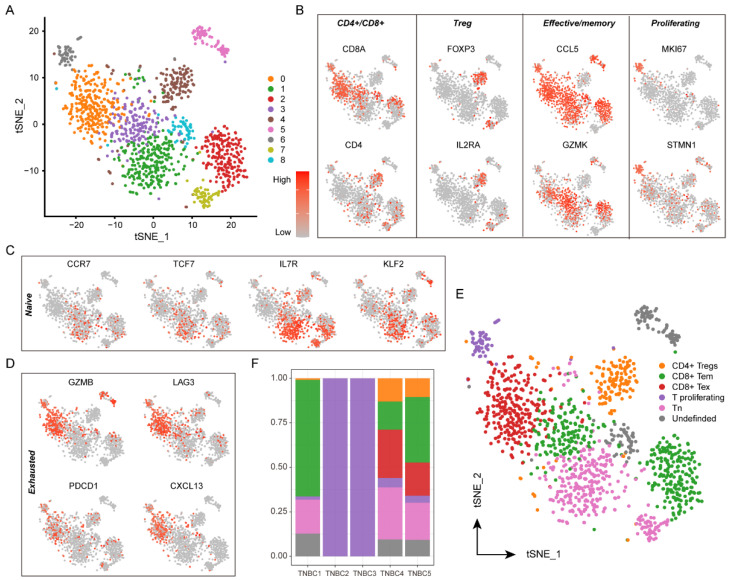
T-cell subpopulations and gene expression in TNBC. (**A**) t-SNE plot of the T cells in the tumors color-coded by the associated cell clusters. (**B**–**D**) t-SNE plot of marker genes for the T-cell subpopulations as indicated, (**B**) CD4^+^/CD8^+^, Treg, effective/memory, and proliferating, (**C**) naïve, and (**D**) exhausted, the shades of red indicating the gene expression levels. (**E**) t-SNE plot of the T cells in the tumors color-coded by the annotated functional T-cell subpopulations. (**F**) Relative proportions of T-cell types for each TNBC patient.

To explore the diversity of T-cells in the TNBC microenvironment, we subclustered T-cells. The analysis resulted in nine distinct cell clusters (Figure 2A). Based on the functional gene marker, we annotated CD4^+^ (cluster 4), CD8^+^ (clusters 0, 2, 3, and 8), regulatory (cluster 4), effective/memory (clusters 0, 1, 2, 3, 5, 6, and 8), and proliferating T-cells (cluster 6) (Figure 2B). In addition, clusters 1 and 7 showed the naïve T-cell characteristics (T_n_ cells), such as the expression of the chemokine-receptor (*CCR7*), interleukin-receptor (*IL7R*), and transcription-factors (*TCF7* and *KLF2*) (Figure 2C). We also found some CD8^+^ T-cells (cluster 0) expressing *PDCD1*, representing activated T cells with exhaustion-like characteristics, based on the expression of the immune-checkpoint (*LAG3* and *PDCD1*), chemokine-receptor (*CXCL13*), and cytotoxic (*GZMB*) markers (Figure 2D). We refer to these as experienced T cells (CD8^+^ T_ex_ cells). Finally, we annotated five functional T-cell subpopulations, including 142 CD4^+^ Tregs, 425 CD8^+^ effective/memory T-cells (T_em_), 275 CD8^+^ T_ex_ cells, 61 proliferative T cells, 322 T_n_ cells, and 73 TRDC+ T cell. (Figure 2E and Figure S3). The obvious individual differences were also observed among the TNBC patients, in which case TNBC1 showed the highest proportion of CD8^+^ T_ex_ cells, cases TNBC4 and TNBC5 were identified with the CD8^+^ T_ex_ cells, and cases TNBC2 and TNBC3 had all of the proliferative T cells in their microenvironment (Figure 2F).

### 3.4. Heterogeneity of Myeloid Cells in the Tumor Microenvironment

**Figure 3:**
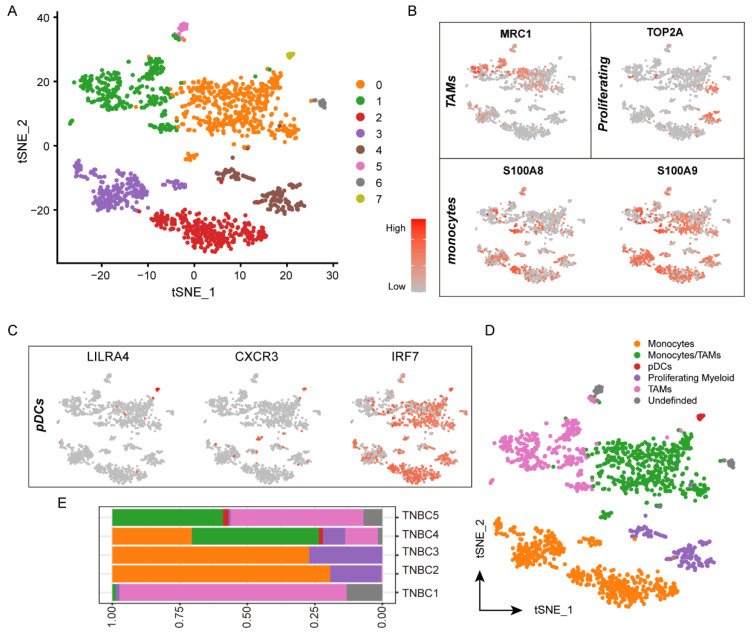
Myeloid-cell subpopulations and gene expression in TNBC. (**A**) t-SNE plot of the myeloid cells in the tumors, color-coded by the associated cell clusters. (**B**,**C**) t-SNE plot of marker genes for the myeloid-cell subpopulations as indicated, (**B**) TAMs, monocytes, and proliferating, and (**C**) pDCs, the shades of red indicating the gene expression levels. (**D**) t-SNE plot of the myeloid cells in the tumors color-coded by the annotated functional myeloid cells subpopulations. (**E**) Relative proportions of myeloid-cell types for each TNBC patient.

To precisely define the function of myeloid cells in TNBC, we subclustered the myeloid cells to characterize the heterogeneity of myeloid cells (Figure 3A). A total of seven clusters were identified (Figure 3B,C), including tumor-associated macrophages (TAMs, clusters 0 and 1), proliferative myeloid cells (cluster 4), monocytes (clusters 0, 2, and 3), and plasmacytoid dendritic cells (pDCs, cluster 7). We annotated five functional myeloid-cell subpopulations, including 529 monocytes, 475 monocytes/TAMs, 338 TAMs, 157 proliferative myeloid cells, and 18 pDCs (Figure 3D). Alternatively, the obvious individual differences also existed in myeloid cells (Figure 3E). However, generally, monocytes/TAMs were identified in all cases.

### 3.5. Malignant Epithelial Cells in TMBC Show a Tendency from Epithelial-Like to Mesenchymal-Like

**Figure 4:**
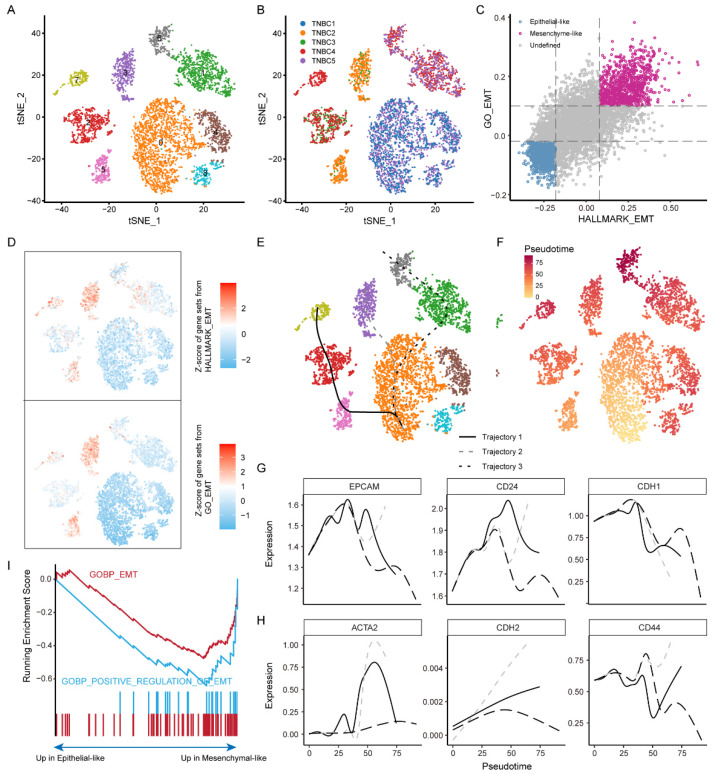
Subpopulations of malignant epithelial cells showing a transition from epithelium-like to mesenchyme-like. (**A**,**B**) t-SNE visualization of TNBC malignant cells. Clusters were annotated for (**A**) cell clusters and (**B**) cancer patients, respectively. (**C**) Scatter plot showing the epithelium-like (blue) and mesenchyme-like (pink) malignant cells. (**D**) The t-SNE map showing TNBC malignant cells was colored by the EMT-score. Color from blue to red reflects epithelial-like to mesenchymal-like process. (**E**,**F**) Pseudotime trajectories for malignant cells of TNBC, color-coded for malignant cell clusters (**E**), pseudotime (**F**). (**G**) Plot of marker genes expression of epithelial cells along the pseudotime trajectory of EMT. (**H**) Same as in (**G**) but for mesenchymal cell. (**I**) GSEA of EMT-related genes among the differentially expressed genes between epithelium-like and mesenchyme-like malignant cells.

As our clustering analysis demonstrated the correlation between the EMT process and malignant epithelial cells, we next sought to decipher the EMT process in the TNBC microenvironment via clustering of all the tumor cells (Figure 4A,B). Subsequently, based on the functional interpretation method of Vision [24] (see the “Section 2”), we scored each cell to attribute their EMT features (Figure 4C). A total of 888 epithelium-like and 846 mesenchyme-like malignant cells were defined, respectively. Despite there being individual differences in malignant epithelial cells among the TNBC patients (Figure 4B), epithelium-like and mesenchyme-like malignant cells were generally found in all patients (Figure 4C,D). In addition, we generated computationally imputed pseudo-time trajectories using the R package slingshot (see the “Section 2”). We considered the epithelium-like malignant cells as the root of the trajectories. We observed three distinct trajectories of malignant epithelial cells (Figure 4E,F). Mesenchyme-like malignant cells were more frequent at the end of these trajectories, which also showed that there were trends of transition from epithelium-like to mesenchyme-like in malignant cells. Further, the marker genes *EPCAM*, *CD24*, *CDH1*, *ACTA2*, *CDH2*, and *CD44* also confirmed the inferred trajectories (especially trajectory two) (Figure 4G,H and Figure S4). Epithelial cell adhesion molecule (*EPCAM*), E-cadherin (*CDH1*), N-cadherin (*CDH2*), and alpha-smooth muscle actin (*ACTA2*) have also been demonstrated to be involved in the EMT process by previous studies [37,38,39]. Alternatively, we performed the gene set enrichment analysis (GSEA) between the epithelium-like and mesenchyme-like malignant cells. The result also supported the positive regulation of the EMT process in TNBC malignant cells (Figure 4I). Taken together, the staged process of EMT is simulated, which will be used to identify key biomarkers for tumor metastasis.

### 3.6. Transcription Factors Are Involved in the EMT Process in TNBC

**Figure 5:**
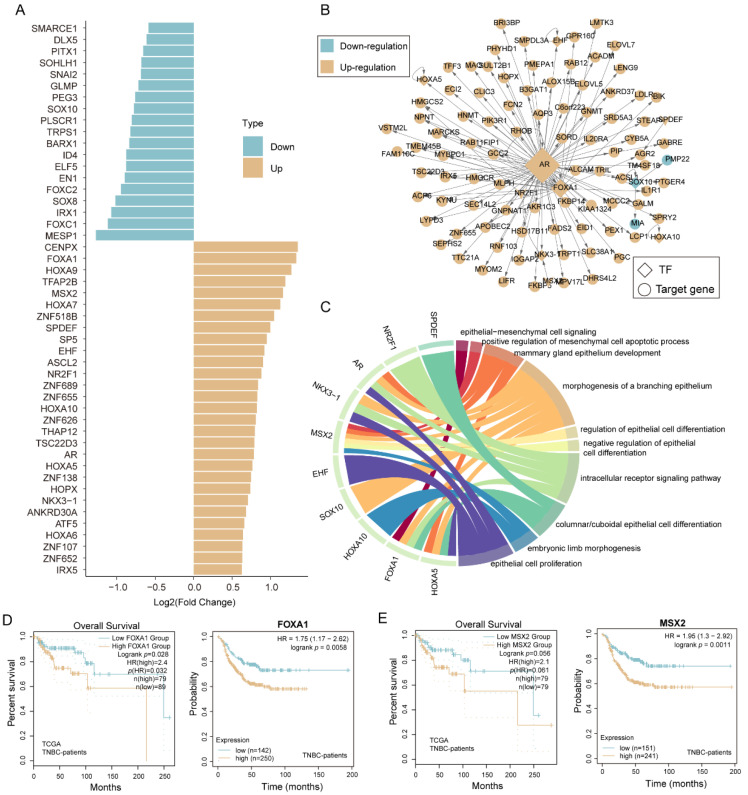
Transcription factor is involved in the EMT process in TNBC. (**A**) Barplot showing differentially expressed TFs between epithelium-like and mesenchyme-like malignant cells. (**B**) TF-downstream gene regulatory network. (**C**) Circosplot showing the relationship between EMT-related TFs and biological pathways. (**D**) Kaplan–Meier curves of TNBC tumor samples stratified by the expression value of *FOXA1*.The left panel shows the patients collected by TCGA and the right panel shows the patients collected by KMPlotter. (**E**) Same as in (**D**) but stratified by the expression value of *MSX2*.

To interrogate the EMT regulators in the TNBC microenvironment, we identified the differentially expressed transcription factors (DE-TFs) between epithelium-like and mesenchyme-like malignant cells (see the “Section 2”). A total of 19 and 29 TFs were significantly down- and up-regulated in mesenchyme-like malignant cells than epithelium-like malignant cells (Figure 5A). We further constructed a TF-downstream gene regulatory network including 10 TFs and 95 target genes [29] (Figure 5B), and annotated the potential biological functions for each TF based on their downstream genes. These TFs were significantly linked to the EMT-related functional terms, such as epithelial-mesenchymal cell signaling, the regulation of epithelial cell differentiation, and morphogenesis of a branching epithelium, etc. (Figure 5C). Across these TFs, we found that overexpressed forkhead box protein A1 (*FOXA1*) and Msh homeobox 2 (*MSX2*) were poor prognosis markers in TNBC patients in both TCGA and external validation cohorts (KMPlotter [40]) (Figure 5D,E). In addition, *FOXA1* and *MSX2* were up-regulated in tumor samples compared to normal samples in TNBC patients (Figure S5A–C) and were experimentally validated to induce the EMT process and tumor metastasis [41,42]. Moreover, independent datasets of TNBC collected from GEO were used to validate the reliability of prognostic markers (Figure S6A,B). These results showed that dysregulated TFs of malignant epithelium cells might play an important role in inducing the EMT process.

### 3.7. EMT-Related Intercellular Communication Patterns in TNBC

**Figure 6:**
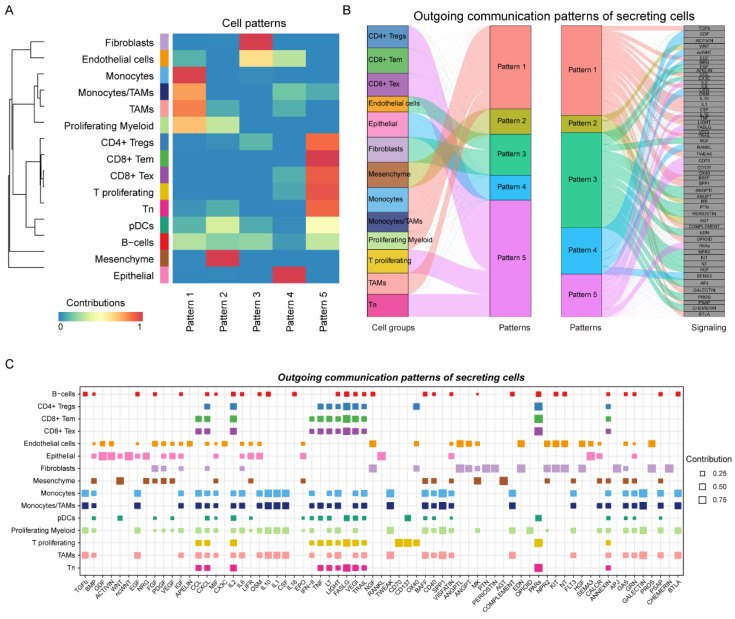
(**A**) Heatmap showing the global five communication patterns calculated by the key signals for subpopulations between cell populations. (**B**) The outgoing communication patterns of secreting cells, which shows the correspondence between the inferred latent patterns and cell groups, as well as signaling pathways. (**C**) Contribution of cell populations to all signaling pathways. The size of the dot is the proportion of the cell population involved in each of the corresponding signal transmissions.

To characterize the cell-cell communications among epithelial-like, mesenchymal-like malignant cells and other microenvironment cells, we integrated the CellChat analysis [31] and detected 623 significant ligand-receptor (L-R) pairs among the 15 cell subpopulations (see the “Section 2”). These ligand-receptor pairs were further categorized into 69 signaling pathways, including TGF-β, non-canonical WNT (ncWNT), TNF, PTN, CXCL, CCL, and KIT pathways. Moreover, the cells were divided into five patterns (Figure 6A). Pattern 1 mainly gathered the cell chat among myeloid cells; pattern 2 was the cell communication dominated by mesenchymal-like malignant cells, including B-cells, pDCs, Tn, TAMs, and proliferating myeloid; pattern 3 exhibited the fibroblasts-related cell-cell interactions; pattern 4 mainly gathered the communications of epithelial-like malignant cells (endothelial cells, CD8^+^ T_ex_ cells, proliferative T-cells, monocytes/TAMs, and TAMs); and pattern 5 showed the crosstalk among T-cells. We also found that pattern 2 expressed the WNT, MK, and AGT signaling pathways, while pattern 4 expressed the GDF, ACTIVIN, NRG, LIFR, OSM, RANKL, and SEMA3 signaling pathways (Figure 6B). Notably, the WNT and RANKL signaling pathways had been demonstrated to be highly associated with the EMT process [43]. In addition, we have studied the changes in the outgoing signaling (Figure 6C). In both pattern 2 and pattern 4, epithelial-like and mesenchymal-like malignant cells were found to communicate with monocytes/TAMs. Specifically, we observed that the TNF signaling pathway was one of the most important outgoing communication patterns secreted by monocytes/TAMs, which had been proven to mediate TNBC growth [44].

### 3.8. TNF Signaling Pathway Mediate the Communications between Monocytes/TAMs and Malignant Epithelial Cells

**Figure 7:**
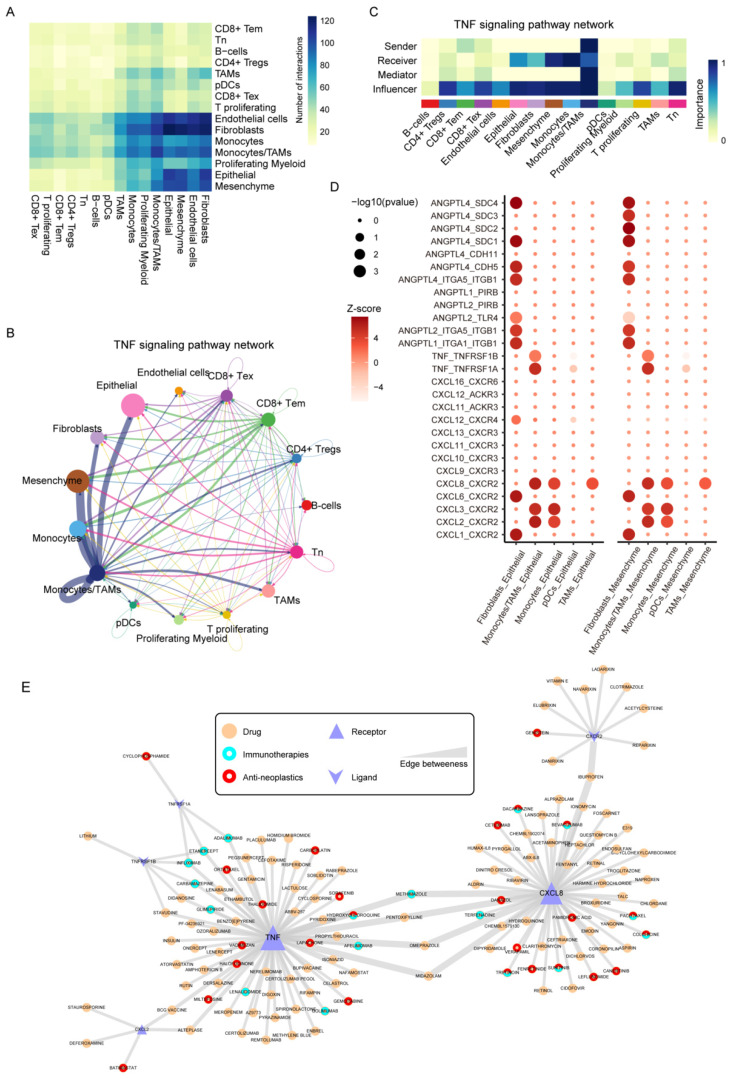
Cell-cell ligand-receptor network analysis. (**A**) Intercellular interactions across microenvironment cells in TNBC. (**B**) Circle plots displaying the inferred network of the TNF signaling pathway. The edge width is proportional to the inferred communication amount. (**C**) Heatmap showing the relative importance of each cell group based on the computed four network centrality measures of TNF signaling. (**D**) Bubble plot showing the ligand-receptor between malignant cells and fibroblasts, monocytes, TAMs, and pDCs. (**E**) Network showing the potential drugs for TNF-related L-R pairs.

Next, we explored the role of signaling pathways in the communications between cell types. We analyzed the interactions between the 15 cell types. There is a large amount of cellular communication between fibroblasts, monocytes/TAMs, epithelial-like cells, and mesenchymal-like cells (Figure 7A). Through functional similarity analysis, 69 signal pathways were clustered into four clusters (Figure S7A,B). Among them, the signal pathway in cluster 3 was mainly related to the proliferation and death of tumor cells. The result showed that multiple TNF interactions were demonstrated among monocytes/TAMs, monocytes, epithelial-like, and mesenchymal-like malignant cells (Figure 7B), in which monocytes/TAMs were the dominant signaling sender, and monocytes, epithelial-like, and mesenchymal-like malignant cells acted as the signaling receivers (Figure 7C). Additionally, CD8^+^ Tem also contributed to the TNF signaling pathway, which revealed the positive feedback of CD8^+^ Tem on tumor invasion. Furthermore, we identified their ligand-receptor interactions (Figure 7D). We found that TNF-TNFRSF1A made a major contribution to the TNF signaling pathway (Figure S7C). Monocytes/TAMs expressed higher levels of tumor necrosis factor (*TNF*), while its receptors *TNFRSF1A* and *TNFRSF1B* were expressed in epithelial-like and mesenchymal-like malignant cells, which could be a key reason for *TNF*-induced EMT process [45,46]. On the other hand, C-X-C motif chemokine ligands (*CXCL2*, *CXCL3*, and *CXCL8*) secreted by monocytes/TAMs could bind to their CXC-chemokine receptor (*CXCR2*), which is expressed by epithelial-like and mesenchymal-like malignant cells. The C-X-C motif chemokine and *CXCR2* had been demonstrated to activate the EMT process [47,48]. These results also indicated that the monocytes/TAMs could induce the EMT process via TNF signaling pathway. Furthermore, to reinforce the clinical implications of these TNF-related ligand-receptor pairs, we constructed drug interactome networks based on the DGIdb database [49] (Figure 7E). Some immunotherapy and anti-neoplastic drugs were identified in the network. Canertinib, an anti-neoplastic drug that could interact with *CXCL8*, had been demonstrated to be effective in inhibiting the growth of breast cancer cells [50]. Infliximab and adalimumab, two immune checkpoint inhibitors that could blockade *TNF*, *TNFRSF1A*, and *TNFRSF1B*, were also included in the network, further highlighting the potential utilities in tumor immunotherapy of TNBC [51]. These results indicated that *TNF*-related L-R pairs exhibited promising values in tumor immunotherapy and anti-tumor strategies in future TNBC treatments.

## 4. Discussion

Intertumoral heterogeneity is one of the major problems limiting the efficacy of diagnosis and therapy [52]. The scRNA-seq technologies support the possibility of identifying the cellular subpopulations and dissecting cell-cell communications in the tumor microenvironment [15,53]. Herein, we utilized the scRNA-seq data to characterize the consistency of the TNBC microenvironment. We found that the individual differences typically existed across our TNBC patients. In addition, intracellular heterogeneity was observed in T-cells, myeloid cells, and malignant epithelial cells. By annotating the highly expressed gene markers, we found that the makers of malignant epithelial cells mainly showed associations with the EMT process. The EMT process is usually considered to be the first step in tumor invasion and metastasis [45], which may also be a key cause of TNBC invasion.

Recent studies have shown that the expression profile of EMT correlates with tumor grade and metastasis in breast cancer [45,54]. Our study also demonstrated that the malignant epithelial cells in TNBC showed transition tendencies from epithelial-like to mesenchymal-like, and several TFs play an important role in the EMT process. We recognized *FOXA1* and *MSX2* as EMT-related TFs, and overexpressed *FOXA1* and *MSX2* were also poor prognosis markers for TNBC. *FOXA1* and *MSX2* were experimentally validated to induce the EMT process and tumor metastasis in other cancers [41,42], indicating that *FOXA1* and *MSX2* may be critical drivers in tumor metastasis of TNBC. The result suggests that EMT-related TFs could be used as potential anti-tumor targets. 

We also characterized the cell-cell communications among epithelial-like, mesenchymal-like malignant cells and other stromal cells based on ligand-receptor pairs. We noticed that monocytes/TAMs could communicate with epithelial-like and mesenchymal-like malignant cells. In addition, *TNF* signaling secreted by monocytes/TAMs contribute significantly to their communication. *TNFRSF1A* and *TNFRSF1B*, as the TNF receptors, were highly expressed in epithelial-like and mesenchymal-like malignant cells, suggesting that the *TNF* signaling pathway may promote tumor metastasis by inducing the EMT process of tumor cells [45,46]. Among the four classical subtypes of breast cancer, only TNBC was identified with significantly higher TNF expression than normal samples (Figure S7C), suggesting that TNF-induced EMT may be specific to TNBC. Previous studies have found that MSX2 acts as a mediator of TNF signaling and is induced by TNF-α in an inflammatory environment [55]. On the other hand, several C-X-C motif chemokine ligands (*CXCL2*, *CXCL3*, and *CXCL8*) could bind to the CXC-chemokine receptor (*CXCR2*), which is expressed by epithelial-like and mesenchymal-like malignant cells. These ligands/receptors could also be potential targets for preventing the EMT process. We also identified some potential drugs which could interact with the TNS-related L-R pairs, including some immunotherapy- and anti-neoplastic- drugs. We observed that bevacizumab could interact with *CXCL8*, which had been demonstrated to be a well-known anti-tumor drug for endometrial cancer and ovarian cancer [56,57]. This also highlighted the potential value of treating TNBC in the future.

## 5. Conclusions

In summary, our study depicted that the intercellular communications could contribute to the EMT process in TNBC. An understanding of the EMT process and crosstalk among microenvironment cells will provide guidance for clinical treatment.

## Data Availability

Publicly available datasets were analyzed in this study.

## Supplementary Materials

The following supporting information can be downloaded at: https://www.mdpi.com/article/10.3390/biom12101478/s1, Figure S1: Quality control of single cell data; Figure S2: Highly variable genes in TNBC tumor cells; Figure S3: Heatmap of the top five genes in malignant cell clusters; Figure S4: T-SNE plot of marker genes in tumor cells; Figure S5: Transcription factor expression in breast cancer; Figure S6: Survival analysis for FOXA1 and MSX2; Figure S7: Cell communications for malignant cells.
