# Supplementary material for: Intercellular Communication Reveals Therapeutic Potential of Epithelial-Mesenchymal Transition in Triple-Negative Breast Cancer
Source: Biomolecules. 2022 Oct 14;12(10):1478. doi: 10.3390/biom12101478 (PMC9599658; doi:10.3390/biom12101478)
Supplement: Supplementary file 1 [file biomolecules-12-01478-s001.zip › biomolecules-1929605-supplementary.pdf]

Intercellular Communication Reveals Therapeutic Potential of Epithelial-Mesenchymal Transition in Triple-Negative Breast Cancer

Liu *et al.*

Supplementary Figures

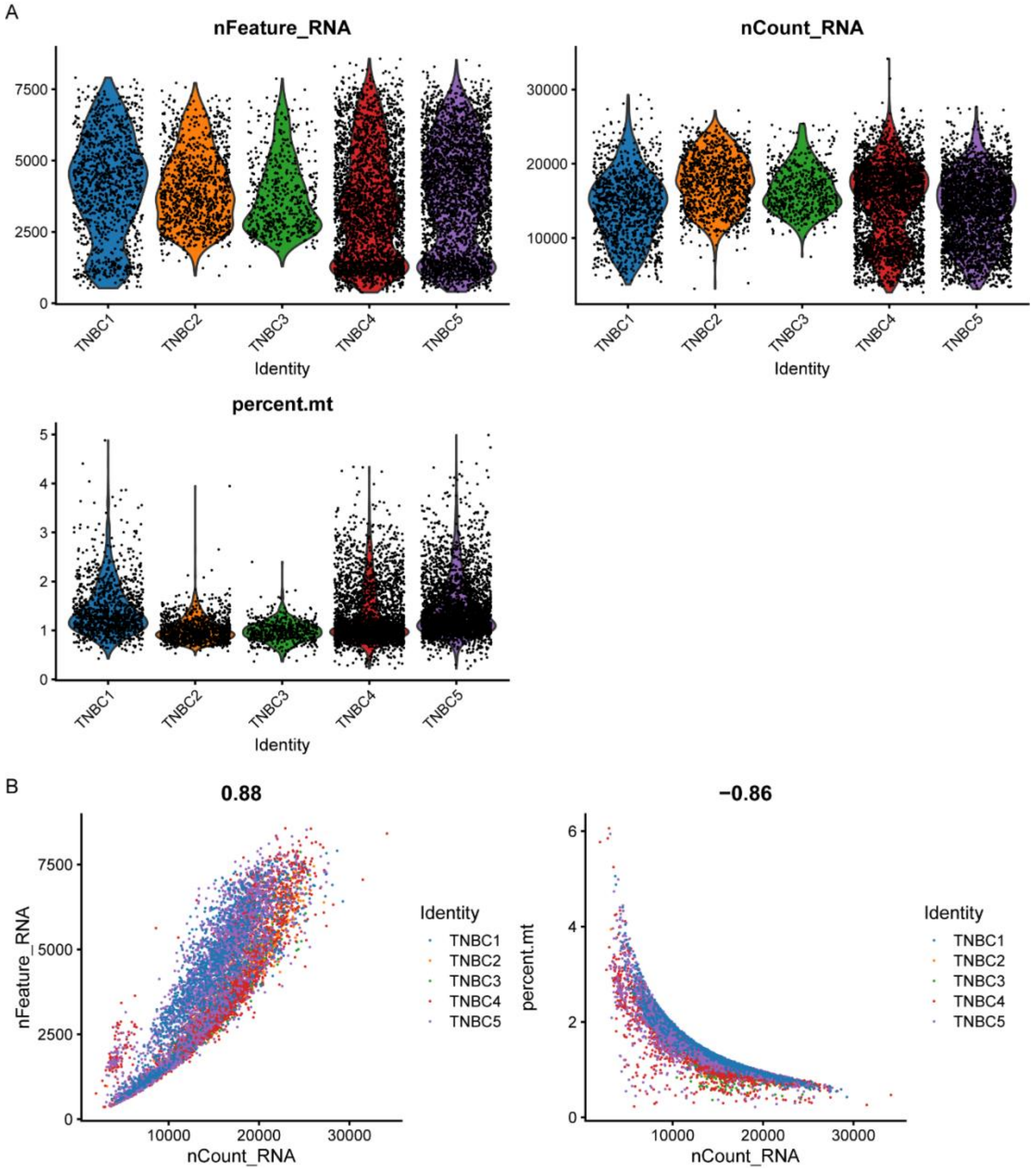

Figure S1. Quality control of single cell data. (A) The violin chart shows the number of genes and count, and the percent of mitochondrial genes detected in each cell. The x-axis represents the patient, and the y-axis represents the number of genes. (B) The scatter plot shows the correlation between sequencing depth and mitochondrial gene sequence.

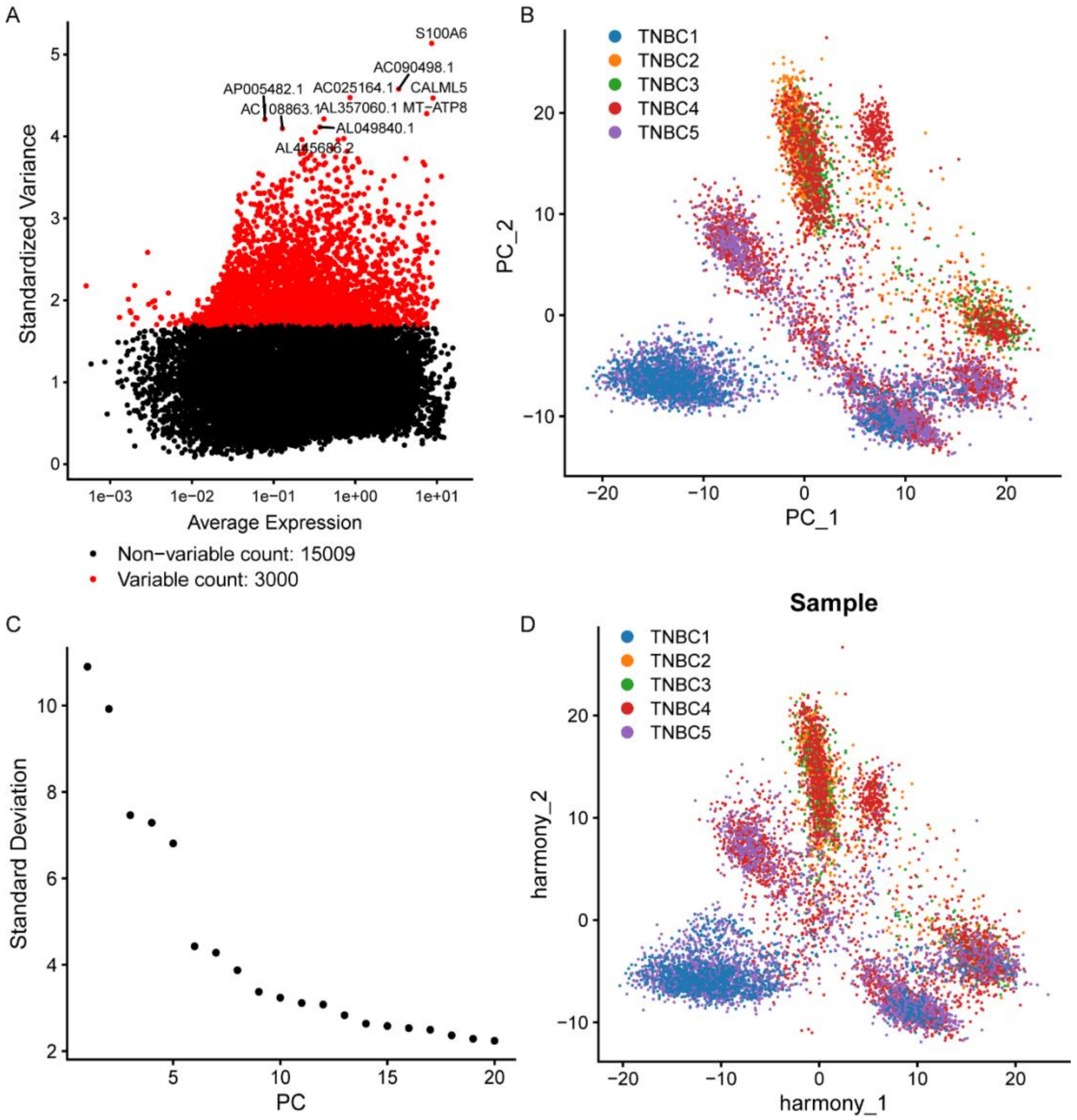

Figure S2. Highly variable genes in TNBC tumor cells. (A) The volcano map shows the expression variation of 18,009 genes, of which 15,009 have low intercellular variation and 3,000 have high variation. (B) Principal component analysis (PCA) shows the first and second principal components of cells. The cells are marked by tissue origin. (C) The elbowplot shows the variation of standard deviation with the number of PCs. (D) Harmony analysis shows the first and second harmony of cells. The cells are marked by tissue origin.

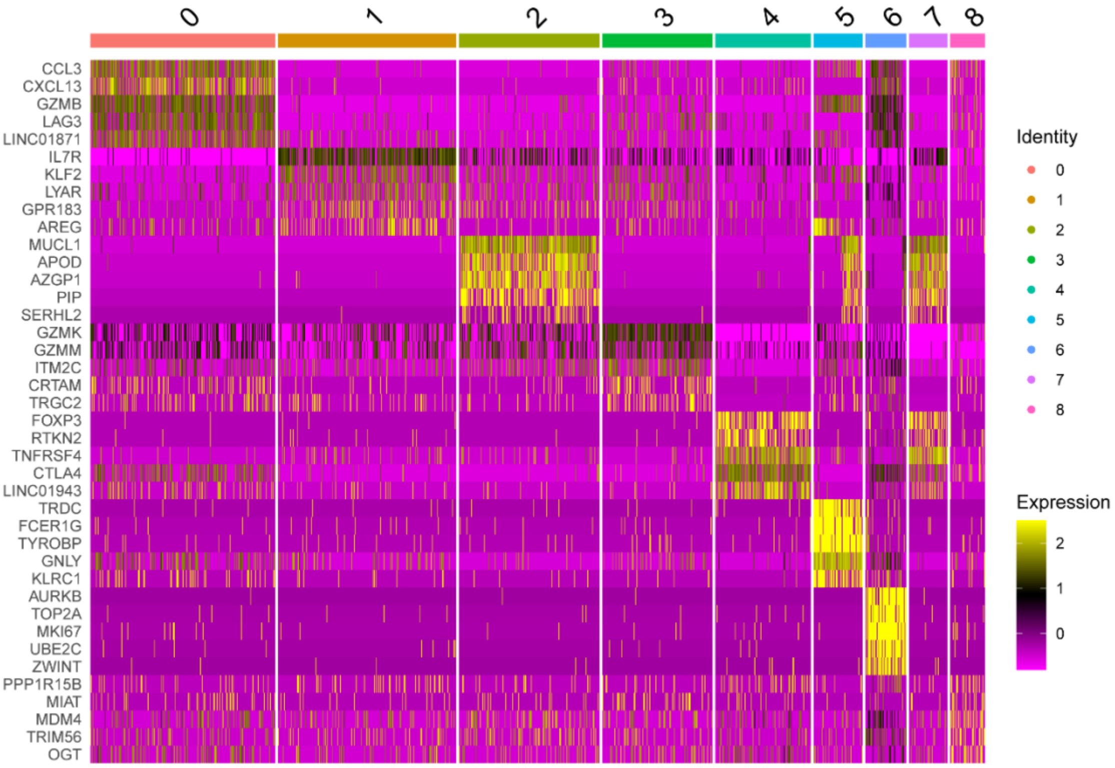

Figure S3. Heatmap of the top five genes in malignant cell clusters.

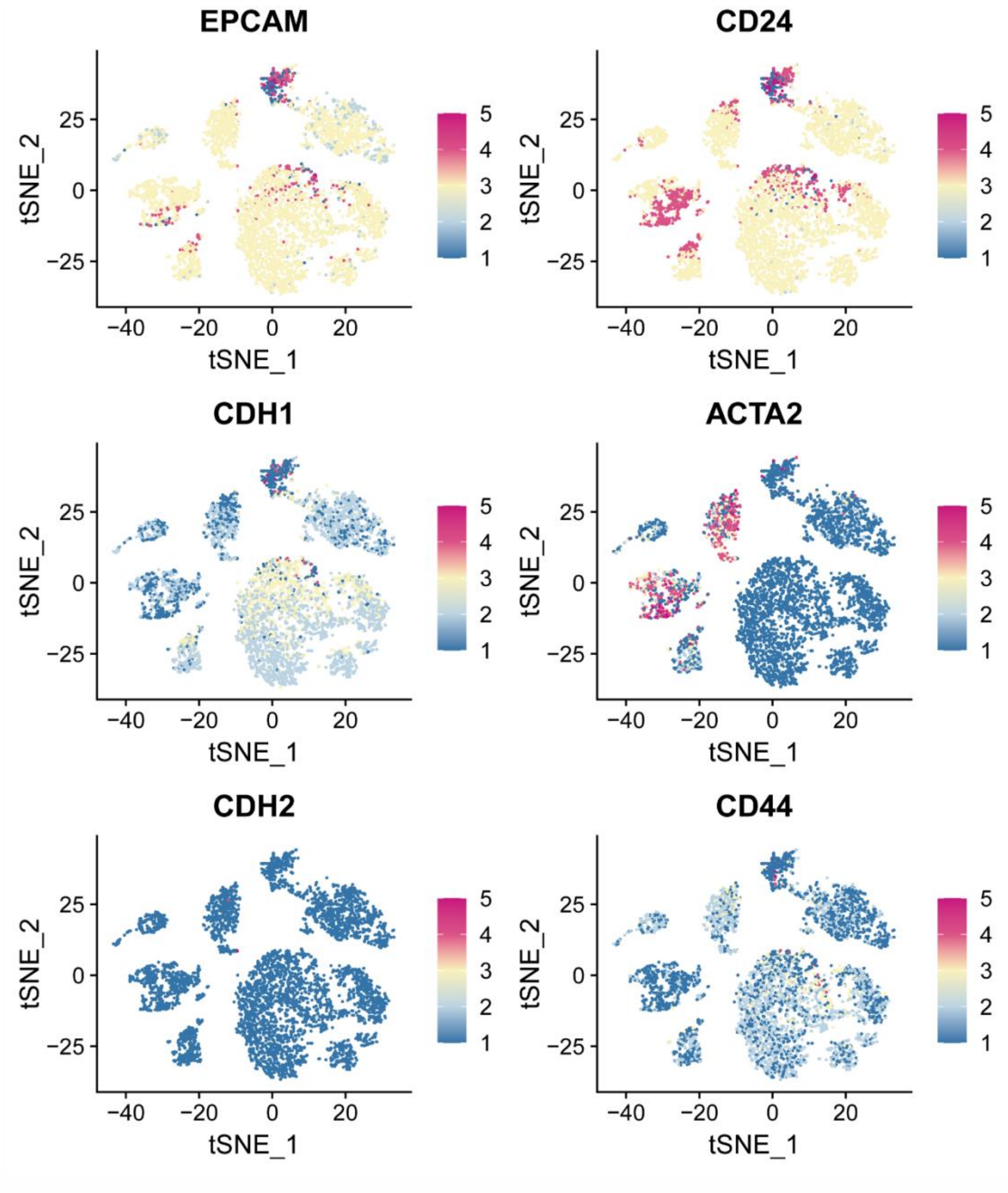

Figure S4. T-SNE plot of marker genes in tumor cells.

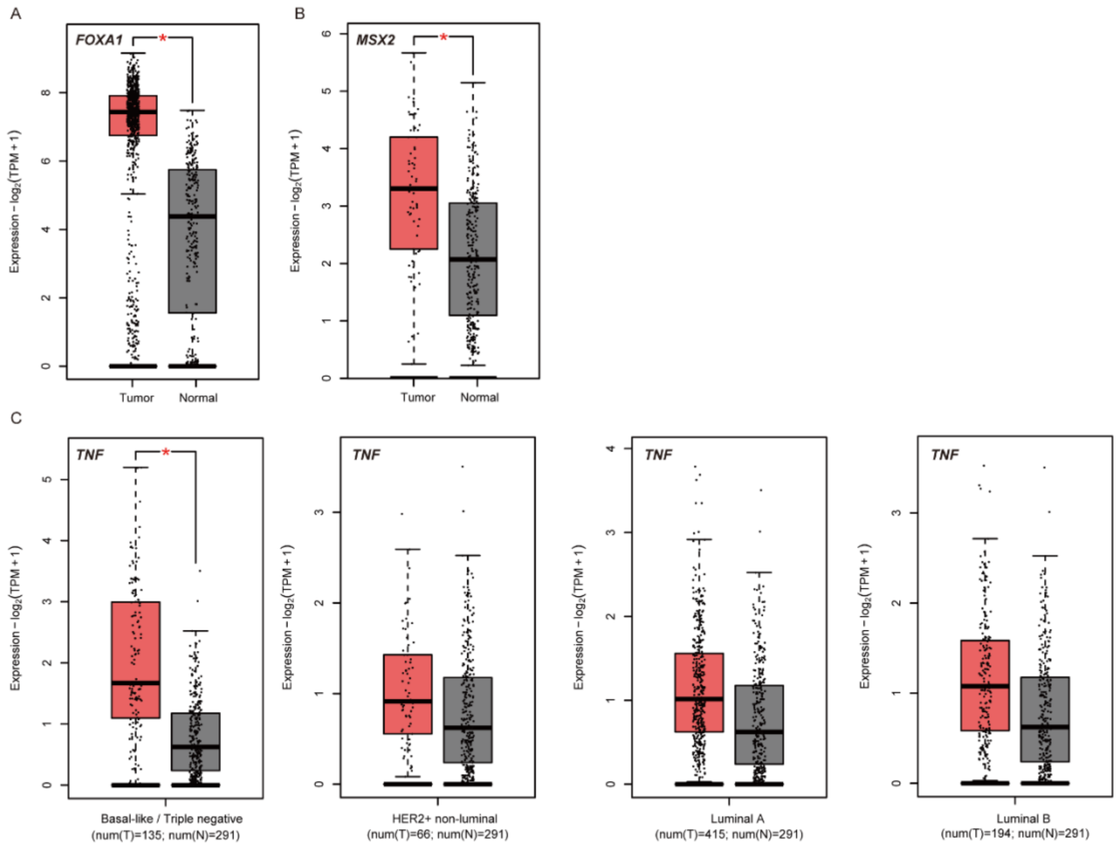

Figure S5. Transcription factor expression in breast cancer. (A-B) The expression of FOXA1 and MSX2 between cancer and normal samples is shown by boxplot. (C) The boxplot shows the expression of TNF between cancer and normal samples from patients with four cancer subtypes.

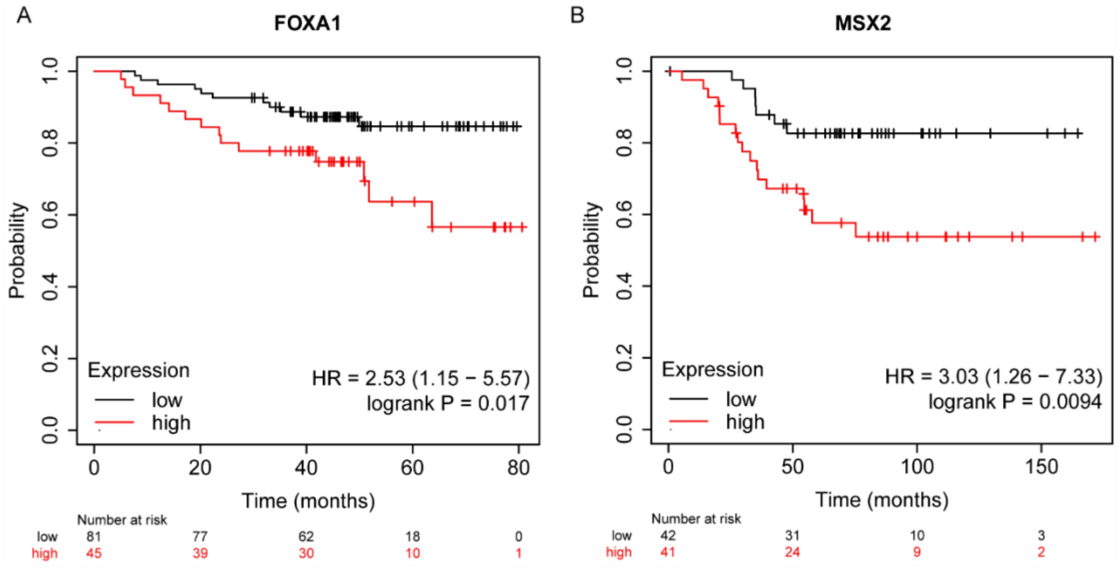

Figure S6. Survival analysis for FOXA1 and MSX2. (A-B) Kaplan-Meier curves of TNBC tumor samples stratified by the expression value of (A) FOXA1 in GSE96058 and (B) MSX2 in GSE58812, respectively.

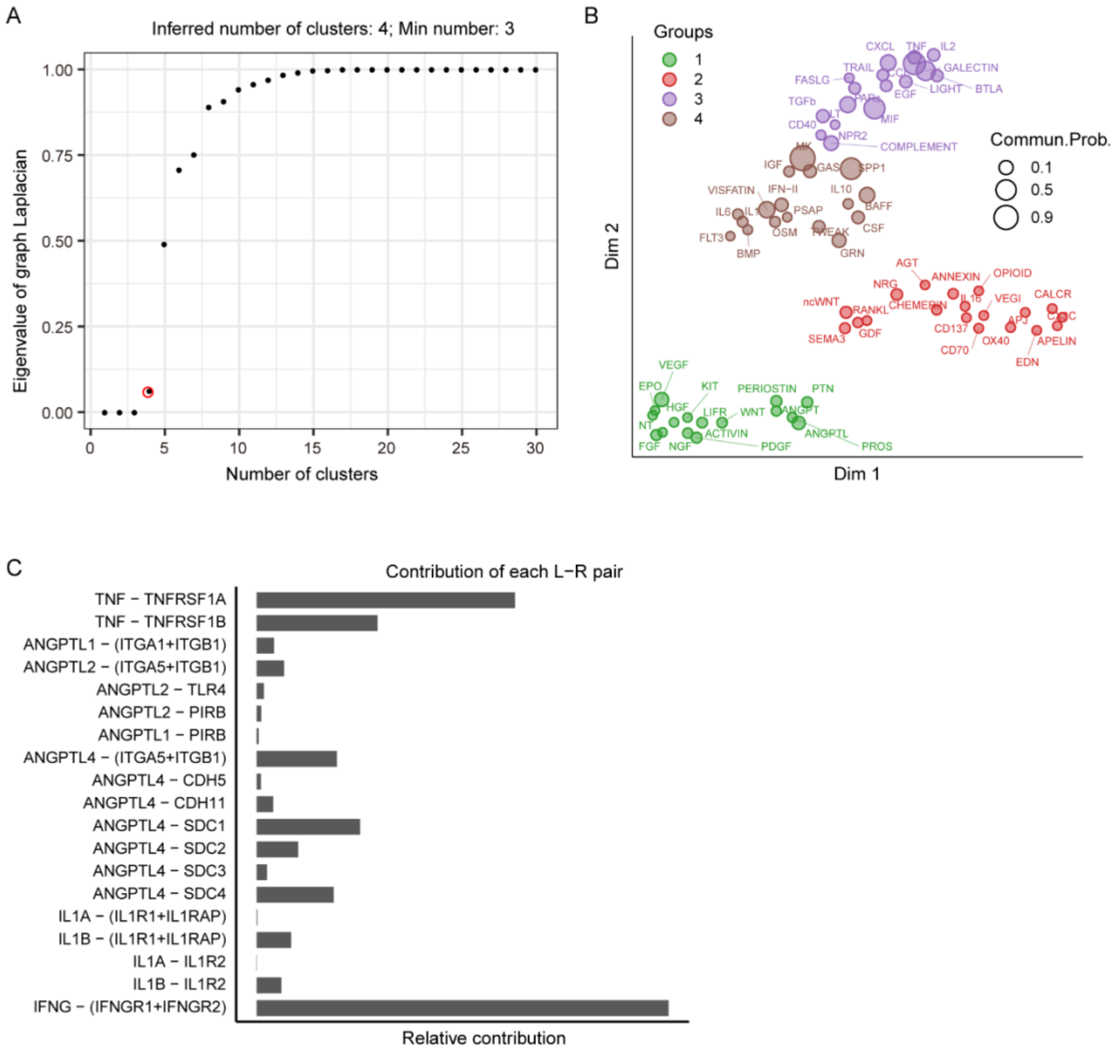

Figure S7. Cell communications for malignant cells. (A) Infer clusters of signal pathways based on functional similarity. The x-axis is the number of clusters, and the y-axis is the eigenvalue of graph Laplacian. (B) The scatter plot shows the dimensionality reduction results of the signal pathways. (C) The bar graph shows the contribution of each ligand-receptor (L-R) pairs to the overall signaling pathway.
